# Quorum Sensing Activity in *Pandoraea pnomenusa* RB38

**DOI:** 10.3390/s140610177

**Published:** 2014-06-10

**Authors:** Robson Ee, Yan-Lue Lim, Lin-Xin Kin, Wai-Fong Yin, Kok-Gan Chan

**Affiliations:** Division of Genetics and Molecular Biology, Institute of Biological Sciences, Faculty of Science, University of Malaya, Kuala Lumpur 50603, Malaysia; E-Mails: robsonee@live.com (R.E.); yanluelim@hotmail.com (Y.-L.L.); kinlinxin@gmail.com (L.-X.K.); yinwaifong@yahoo.com (W.-F.Y.)

**Keywords:** matrix-assisted laser desorption ionization time-of-flight (MALDI-TOF), mass spectrometry (MS), cell-to cell communication, triple quodruopole liquid chromatography mass spectrometry (LC-MS/MS), *N*-octanoyl homoserine lactone (C8-HSL)

## Abstract

Strain RB38 was recovered from a former dumping area in Malaysia. MALDI-TOF mass spectrometry and genomic analysis identified strain RB-38 as *Pandoraea pnomenusa*. Various biosensors confirmed its quorum sensing properties. High resolution triple quadrupole liquid chromatography–mass spectrometry analysis was subsequently used to characterize the *N*-acyl homoserine lactone production profile of *P. pnomenusa* strain RB38, which validated that this isolate produced *N*-octanoyl homoserine lactone as a quorum sensing molecule. This is the first report of the production of *N*-octanoyl homoserine lactone by *P. pnomenusa* strain RB38.

## 1. Introduction

*Pandoraea* is a Gram-negative rod shape proteobacteria of the Burkholderiaceae family that is often isolated from sputa of cystic fibrosis patients and soil [1]. Initially documented by Coenye and co-workers [1], this genus is primarily represented by four cystic fibrosis (CF) isolates, namely: *Pandoraea pnomenusa*, *Pandoraea pulmonicola*, *Pandoraea apista* and *Pandoraea sputorum* [2] which are often misclassified as their neighboring organisms (*Burkholderia* and *Ralstonia*) or even described as non-fermentative Gram-negative bacteria [3–5]. *P. pnomenusa* in particular, is recognized as a causing agent for bacteremia, multiple organ failure and it also causes mortality [6]. *P. pnomenusa* has been reported as a multi-drug resistant pathogen [4].

Quorum sensing (QS) bacteria produce diffusible signaling molecules to communicate with one another as the bacterial population increases in order to regulate gene expression in unison [7–9]. Gram-negative bacteria have the ability to secrete *N*-acylhomoserine lactones (AHLs) as their autoinducers. QS modulates a battery of bacterial physiological activities which include swimming, swarming, sporulation, motility, bioluminescence, biofilm, antibiotic biosynthesis and plasmid conjugal transfer by coordinating the gene expression of a population [7,10,11]. The latest discovery is the documentation of QS properties in *Pandoraea* sp. [12]. Because of the importance of AHLs [13], this study aims to characterize the AHL secreted by *P. pnomenusa* RB-38 using triple quadrupole mass spectrometry (LC-MS/MS) in order to gain a better understanding of the QS activity of this bacterium.

Here, we present the QS properties of *P. pnomenusa* RB38. Its identity was confirmed *via* MALDI-TOF MS analysis and 16S rDNA sequencing. Different AHL biosensors were used for preliminary screening of AHLs and subsequently the *P. pnomenusa* RB38 AHL profile was characterized by LC-MS/MS analysis. To our best knowledge, this is the first documentation of the QS profile in the species *P. pnomenusa*.

## 2. Experimental Section

### 2.1. Bacterial Growth Conditions

All strains (Table 1) were cultured aerobically at 28 °C in Luria-Bertani (LB) broth or LB agar with exception for *Escherichia coli* [pSB401], which was incubated at 37 °C. The antibiotic tetracycline (20 μg/mL) was supplemented into the growth media when necessary.

### 2.2. Strain Isolation

In the search for QS bacteria from a dumping ground (subsurface of 10 cm from the top soil) in Malaysia (GPS coordinate of N03'00'12.1, E101' 39'33'1), strain RB38 was isolated for further studies. The sampling site was 61 m above sea level. To isolate soil bacteria, we used KGm [12,17]. The soil sample was inoculated into KGm and underwent four enrichment cycles using a reported method [12,17]. Single pure colonies of soil bacteria were obtained by serial dilution streaking on LB agars.

### 2.3. Bacterial Identification

MALDI-TOF MS analysis and 16S rDNA nucleotide sequencing was performed as described previously [12,18–20]. Nucleotide comparison was performed using the BLASTN program in the NCBI database and the phylogenetic tree was constructed using Molecular Evolutionary Genetic Analysis (MEGA).

### 2.4. Detection of Short Chain AHLs Productions

AHL screening was performed using the CV026 biosensor. *P. pnomenusa* RB38 and the experimental control strains were cross-streaked with CV026. Purple pigmentation after 24 h indicates QS activity.

### 2.5. AHL Extractions

*P. pnomenusa* RB38 was cultured in 100 mL LB broth supplemented with MOPS buffer. Growth media were buffered to acidic condition at pH 5.5 to prevent lactonolysis because AHLs are unstable in alkaline conditions [21,22]. Triplicate AHL extractions were performed twice with 150 mL of acidified ethyl acetate. The upper non-polar immiscible solvent layer was then transferred into a sterile beaker and the other layer was discarded. AHL extracts were dried under sterile conditions. Extracts from LB broth (with no inoculum) served as negative control.

### 2.6. Bioluminescence Assay

Planktonic culture of *E. coli* biosensor was adjusted to OD_600 nm_ of 0.1 using sterile LB broth as diluent. The diluted biosensor cells were used to dissolve AHL extracts and the mixtures transferred to microtitre wells in a 96-well microtitre plate. The luminescence intensity and OD_495 nm_ were determined and recorded simultaneously at 60 min intervals for the duration of 24 h in Infinite M200 luminometer (Tecan, Männerdorf, Switzerland). The bioluminescence assay result was determined as relative light unit per OD_495 nm_ (RLU/OD_495 nm_) *vs.* time [12,23,24].

### 2.7. Determination of AHL by LC-MS/MS

AHL extracts were resuspended with 100 µL acetonitrile and analyzed as described previously [12,17–19] using an Agilent 1290 Infinity LC system (Agilent Technologies Inc., Santa Clara, CA, USA) coupled with an Agilent ZORBAX Rapid Resolution High Definition SB-C18 Threaded column (2.1 mm × 50 mm, 1.8 µm particle size). Electrospray ionization (ESI) with jet-stream positive mode was used as the ion source and detection of *m*/*z* 102 product ion was performed using precursor ion scan mode. Data analysis was conducted using the Agilent Mass Hunter software.

### 2.8. Nucleotide Sequence Accession Number

We have deposited the 16S rDNA nucleotides sequences of *P. pnomenusa* RB38 into NCBI and was assigned GenBank accession No. KJ507404. For molecular analysis, we obtained the other 16S rDNA nucleotides sequences from GenBank database.

## 3. Results and Discussion

### 3.1. Isolation and Identity Classification of Soil Bacterium Strain RB38

**Figure 1.**
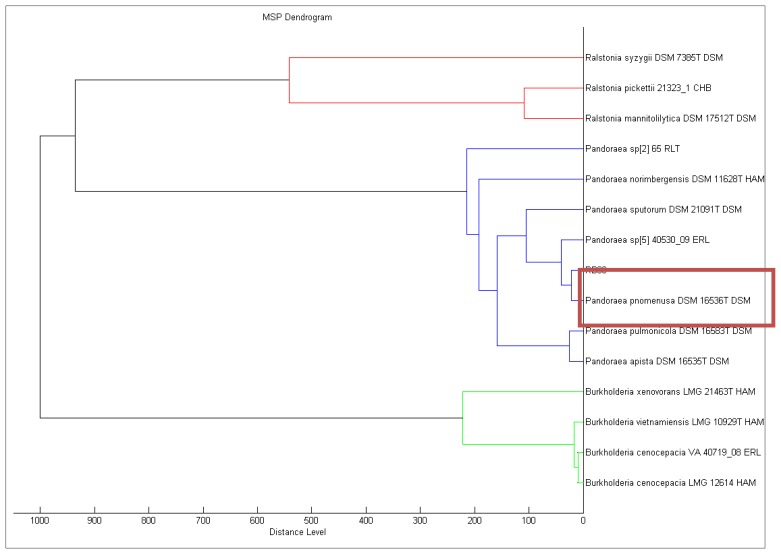
Dendrogram of isolate RB38. MALDI-TOF analysis confirmed isolate RB38 clusters with *Pandoraea pnomenusa*.

KGm medium supplemented with 3-oxo-C6-HSL (50 mM final concentration, Sigma-Aldrich, St Loius, MO, USA) was used for the soil bacteria enrichment process [12,17]. Within 48 h post-incubation, viable cell count showed that microbial growth occurred in KGm medium. Several distinct morphological colonies of bacteria were selected and a succession of streaking was conducted to obtained pure colonies. A strain labeled as RB38 which was identified as *P. pnomenusa* MALDI-TOF (Figure 1) was selected for further analysis.

**Figure 2.**
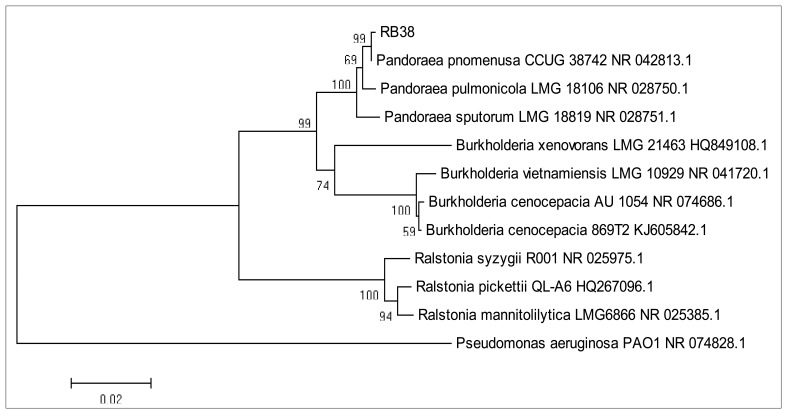
Evolutionary tree of 16S rDNA nucleotide sequences of isolate RB-38 with its closest neighbours using the Neighbour-Joining method.

Biochemical identification using the Microbial Identification System has often misclassified *Pandoraea* as *Burkholderia* and *Ralstonia* species due to the similarity of biochemical activities between these two genera. Thus, 16S rDNA sequence analysis was performed to further confirm the identity of isolate RB38 (Figure 2). Phylogeny of 16S rDNA nucleotide sequences of isolate RB38 with its closest neighbours was constructed using Neighbour-Joining algorithm [25]. The evolutionary distances were computed using the Maximum Likelihood method [26] and expressed as the number of base changes per site. The analysis involved seven nucleotide sequences datasets. There were a total of 1,394 positions in the final dataset. For evolutionary analyses, we used MEGA 5 as reported elsewhere [27].

16S rDNA analysis has been reported as a better option if there is difficulty in identifying a pathogen as *B. cepacia* complex or *Ralstonia* species [6]. Molecular analysis also confirmed the data from MALDI-TOF for microorganism identification [17]. In 2002, Coenye has documented a better method of detection of *Pandoraea* using restriction fragment length polymorphism (RFLP) analysis and nucleotide sequencing of a house-keeping gene (*gyr* B) for better resolution to distinguish closely-related species in the genus of *Pandoraea* [28].

### 3.2. AHL Screening

**Figure 3.**
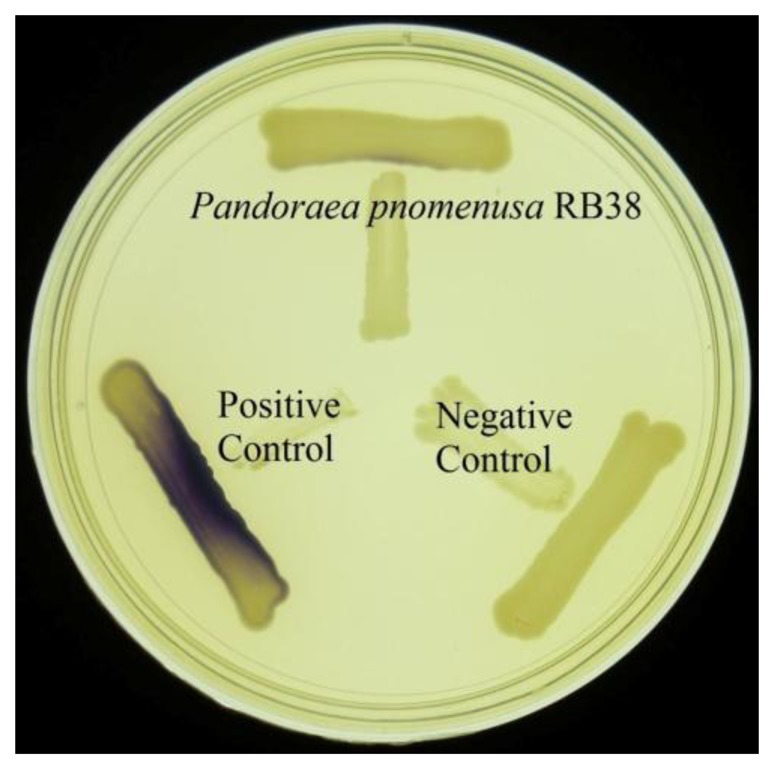
AHL screening of *P. pnomenusa* RB38. Biosensors *C. violaceum* CV026 was streaked adjacent to the test strains. Note that purple pigment shows quorum sensing activity. *Erwinia carotovora* GS101 (Positive control) and *Erwinia carotovora* PNP22 (Negative control) were included as controls.

**Figure 4.**
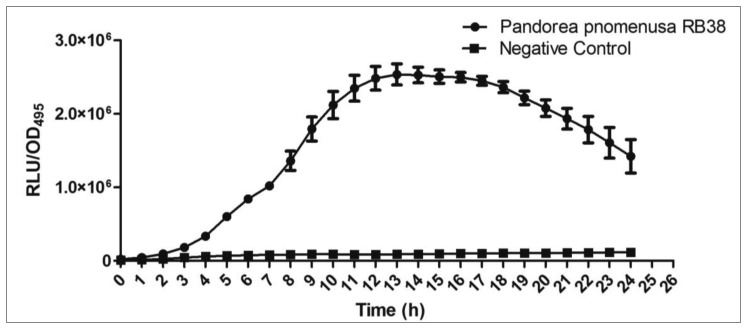
Bioluminescence assay for production of AHLs by *P. pnomenusa* RB38. Each dot symbolizes the mean results of triplicate experiments. Negative control was included which is an extract from uninoculated LB broth.

Rapid screening of AHL production by soil isolates was conducted using the *C. violaceum* CV026 biosensor [14]. Formation of purple pigmentation in *C. violaceum* CV026 after a 1-day incubation with *P. pnomenusa* RB38 indicated detection of short chain AHLs produced by *P. pnomenusa* RB38 (Figure 3). The *C. violaceum* CV026 biosensor is capable of detecting short chain AHLs in the range of *N*-butanoyl homoserine lactone (C4-HSL) to *N*-octanoyl homoserine lactone (C8-HSL). Further investigation was performed in bioluminescence assay using *lux*-based *E. coli* [pSB401] biosensor with a detection range of C4-HSL to C8-HSL [16]. Luminescence bioassay involving *E. coli* [pSB401] suggested production of short chain AHLs by *P. pnomenusa* RB38 (Figure 4).

### 3.3. LC-MS/MS Analysis of AHLs

**Figure 5.**
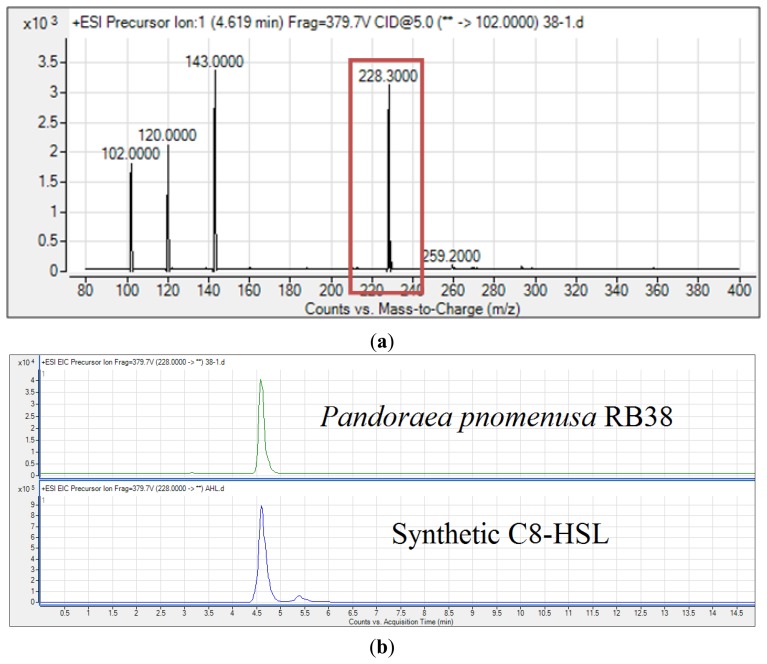
Mass spectrometry analysis of AHLs produced by *P. pnomenusa* RB38. Mass spectrum shows that *P. pnomenusa* RB38 produced C8-HSL (**a**); Retention time of C8-HSL from spent supernatant of *P. pnomenusa* RB38 is the same as the retention time of synthetic C8-HSL (**b**).

There are many reports on the regulation of virulence factors by QS mechanisms in CF pathogens such as *Pseudomonas aeruginosa* and *Burkholderia* spp. [29–32]. Thus, it is important to ensure *P. pnomenusa* produces AHLs. To identify the AHL profile of *P. pnomenusa* RB38 in the spent supernatant of *P. pnomenusa* RB38, our MS data validated the presence of C8-HSL (*m*/*z* value of 228.3, retention time: 4.602 min; abundance: 3342.7; abundance %: 95.71) in the supernatant of this strain (Figure 5). The presence of three product fragment ions with *m*/*z* values of 102, 120 and 143 was also detected, thus confirming unequivocal presence of this AHL.

## 4. Conclusions

Here, we report *P. pnomenusa* RB38 produced C8-HSL. To our best knowledge, this is the first documentation of short chain AHL production in the species *P. pnomenusa*.

## Figures and Tables

**Table 1.** Bacteria strains used in this study.

Bacterial Strains Used	Descriptions	Source/Reference
*Pandoraea pnomenusa* RB38	Isolated from ex-landfill site	This study
*Chromobacterium violaceum* CV026	AHL biosensor that synthesizes purple pigment in detection of short chain *N*-acyl-side chain length of four to eight carbons.	[14]
*Erwinia carotovora* GS101	Positive control of the CV026 AHL detection	[15]
*Erwinia carotovora* PNP22	Negative control of the CV026 AHL detection	[15]
*Escherichia coli* [pSB401]	Short chain AHLs biosensor used for the bioluminescence assay, Tet^R^	[16]
